# Germline Variants in Bladder and Upper Tract Urothelial Cancers: Prevalence and Clinical Context in a Large Testing Registry

**DOI:** 10.1016/j.euros.2026.02.010

**Published:** 2026-02-23

**Authors:** Steven M. Monda, Eugene Oh, Patrick J. Lewicki, Samuel D. Kaffenberger, Tobias Else, Zachery R. Reichert, Irene Tsung, Khurshid R. Ghani, Charles B. Nguyen, Rob Humble, Matthew J. Schiewer, Robert Finch, Simpa Salami, Elena M. Stoffel, Todd M. Morgan, Thenappan Chandrasekar, Udit Singhal

**Affiliations:** a Department of Urology, University of Michigan, Ann Arbor, MI, USA; b School of Medicine, University of Michigan, Ann Arbor, MI, USA; c Division of Genetic Medicine, University of Michigan, Ann Arbor, MI, USA; d Division of Hematology and Oncology, University of Michigan, Ann Arbor, MI, USA; e Department of Medical Oncology, City of Hope Comprehensive Cancer Center, Duarte, CA, USA; f Department of Pathology, University of Michigan, Ann Arbor, MI, USA; g Myriad Genetics, Salt Lake City, UT, USA; h Division of Gastroenterology, University of Michigan, Ann Arbor, MI, USA; i Department of Urology, University of California Davis, Sacramento, CA, USA

**Keywords:** Germline testing, Urothelial cancer, Upper tract urothelial cancer, Lynch syndrome, Homologous recombination repair, Mismatch repair, Hereditary cancer syndromes, Germline variant, Bladder cancer

## Abstract

**Background and objective:**

The prevalence and clinical history of germline variants in bladder cancer and upper tract urothelial cancer (UTUC) remains incompletely defined, particularly regarding mismatch repair (MMR) and homologous recombination repair (HRR) variants. This study aims to evaluate the prevalence of germline variants in patients with bladder cancer and/or UTUC referred for germline testing, and to report the personal and family cancer histories of patients with MMR (Lynch syndrome) and HRR variants.

**Methods:**

We retrospectively analyzed 3561 urothelial cancer patients (3130 with bladder cancer only, 370 with UTUC, and 61 with both) who underwent germline testing between 1996 and 2025 at Myriad Genetics. We describe the prevalence of pathogenic/likely pathogenic germline variants in patients with UTUC and bladder cancer, and characterize the personal and family cancer histories of patients with MMR (*MSH2*, *MSH6*, *MLH1*, and *PMS2)* and HRR (*BRCA1* and *BRCA2,* among others) variants, comparing with an institutional cohort of MMR carriers (*n* = 35; 2012–2025). We also assess the proportion of MMR variant carriers who would be referred for testing under existing guidelines.

Key findings and limitations

In this cohort, 702 (20%) patients had pathogenic/likely pathogenic germline variants, including 328 patients with MMR and 298 patients with HRR variants. MMR variants were more common in UTUC, with 27% (*n* = 101) harboring an *MSH2* variant compared with 7.6% (*n* = 109) of bladder cancer patients (*p* < 0.001). However, conversely, 58% (*n* = 109) of patients with an *MSH2* variant had bladder-only disease. Prior nonurothelial cancers occurred in 54–63% of MMR and 35–41% of HRR carriers.

**Conclusions and clinical implications:**

Lynch syndrome is common in patients with UTUC; yet, many carriers present with bladder cancer alone. Personal and family cancer histories frequently precede urothelial cancer, underscoring the need for routine germline testing in UTUC and consideration of broader testing across urothelial cancer.

**Patient summary:**

Inherited genetic variants, especially those associated with Lynch syndrome, are common in patients with ureter and renal pelvis urothelial cancer. However, many patients with these variants have urothelial cancer of the bladder only. Broader genetic testing, particularly in those with a suggestive personal or family cancer history, may help identify patients at risk who might otherwise be missed.

## Take Home Message

Lynch syndrome is common in patients with upper tract urothelial cancer, but many affected individuals present with urothelial cancer of the bladder alone. Broader germline testing should be considered in patients with urothelial cancer, including all patients with upper tract urothelial cancer and those with a personal or family history of associated cancers, regardless of urothelial cancer site.

## 1 Introduction

The landscape of germline variants in urothelial carcinoma is not well defined. Despite known associations between Lynch syndrome and urothelial cancer, especially in upper tract urothelial cancer (UTUC), as well as tentative associations with certain homologous recombination repair (HRR) variants, the rate of pathogenic and likely pathogenic variants in urothelial cancer and the cancer histories of patients with these variants is unclear.

Lynch syndrome is caused by germline pathogenic variants in any of four DNA mismatch repair (MMR) genes (*MSH2*, *MSH6*, *MLH1*, and *PMS2*). Tumorigenesis typically follows somatic inactivation of the second allele leading to MMR deficiency and a wide variety of cancers, including urothelial cancer [1]. These tumors are characteristically microsatellite instability high (MSI-high) and often responsive to immune checkpoint inhibition, which has received broad regulatory approval in this setting [2].

With improved protocols for surveillance and early treatment, mortality from colon and endometrial cancers in patients with Lynch syndrome has decreased. In this population, urothelial cancers are now emerging as a leading cause of mortality [3]. For instance, in the Prospective Lynch Syndrome cohort, *MSH2* carriers undergoing regular colonoscopy had nearly equivalent mortality from urothelial and colon cancers [4].

Germline pathogenic variants can also affect a wide variety of genes involved in HRR, most commonly *BRCA1* or *BRCA2.* These HRR variants, leading to homologous recombination deficiency, are associated with a variety of cancers, notably breast, ovarian, and prostate cancers, and lend sensitivity to poly(adenosine diphosphate–ribose) polymerase (PARP) inhibition [5], [6].

Given the clinical implications of identifying germline variants—including cascade testing, cancer screening, and a growing number of targeted therapies—we sought to describe the proportion of pathogenic and likely pathogenic variants, particularly in MMR and HRR genes, in a large cohort of patients with bladder cancer and UTUC referred for germline testing. We also describe the personal and family cancer histories in those with germline MMR (Lynch syndrome) and HRR variants, to provide insights into the cancer chronology and characteristics of these patients. We also report the proportion of patients with Lynch syndrome that would be referred for testing under current guidelines. Lastly, we compare these findings with an internal cohort of patients with Lynch syndrome and urothelial cancer seen at the University of Michigan.

## 2 Patients and methods

This was a retrospective cohort study of data from a cohort of patients who were referred and received germline testing between 1996 and 2025 at Myriad Genetics and had a reported history of urothelial cancer. All patients with completed germline testing and a reported personal history of ureteral, bladder, and/or renal pelvis cancer at any time were included. Patients were grouped as those with bladder cancer only and UTUC, which included ureteral and renal pelvis cancers. Given the tendency of UTUC to seed the bladder, patients with both UTUC and bladder cancer were grouped in the UTUC cohort. All patients were deidentified, and exemption for the analysis of the Myriad cohort was obtained through the University of Michigan Institutional Review Board (HUM00270233). Strengthening the Reporting of OBservational studies in Epidemiology (STROBE) guidelines were adhered to in this retrospective descriptive cohort study [7].

Our first aim was to describe the proportion of germline variants identified in a population that received germline testing and had a personal history of urothelial cancer. Only pathogenic and likely pathogenic variants were considered. All likely pathogenic variants were further corroborated within ClinVar as likely pathogenic or pathogenic, accessed August 2025 [8]. Variants of uncertain significance were excluded and not used in our analysis. *MUTYH* carriers were included in the analysis. To account for variable panel sizes (ie, some panels testing only a subset of genes), variant prevalence for each gene was calculated as the number of carriers of that gene divided by the number of patients tested for that gene. Variant prevalence was compared between patients with bladder cancer only and those with UTUC. Fisher’s exact test was used to for these comparisons, assessing MMR (*MSH2*, *MSH6*, *MLH1*, and *PMS2*) and HRR (specifically *BRCA1* and *BRCA2*) genes.

Our second aim was to describe the personal and family cancer histories in those with germline variants in MMR and HRR genes. Consistent with the recommendations from the European Hereditary Tumor Group, the four MMR genes (*MSH2*, *MSH6*, *MLH1*, and *PMS2*) were considered separately [3]. In our cohort, all eight patients with deletions in *EPCAM* also had pathogenic *MSH2* variants and were thus analyzed within *MSH2*. We also assessed HRR patients, grouped as those with *BRCA1*, *BRCA2*, and other HRR variants (*CHEK2, ATM, PALB2, BRIP1*, *BARD1*, *RAD51C*, and *RAD51D*) separately.

Patient records were queried for all prior malignancies (excluding benign polyps). For skin cancers, only sebaceous carcinomas and melanomas were considered. Sebaceous carcinomas were assessed given their strong and specific association with Lynch syndrome, specifically *MSH2* loss. Separate family cancer histories were also reported for these patients. First-degree family cancer histories were also reported, which included parents, children, and siblings. Sex at birth and ancestry were self-reported, and multiple ancestries could be selected.

To assess the proportion of patients who would be referred to germline screening based on the European Association of Urology (EAU) UTUC guidelines, we reported the percentage of patients with UTUC who would test positive for these revised Amsterdam II criteria [9]. Notably, current bladder cancer guidelines are not explicit on who should be referred for germline testing, so these same EAU guidelines were also applied to the bladder cancer–only patients, noting that these patients would fall outside of the UTUC guidelines given their site of disease.

To corroborate the clinical history of patients with urothelial cancer and Lynch syndrome, we identified an internal institutional cohort of patients with Lynch syndrome and urothelial cancer. We queried encounters at the University of Michigan urology, medical oncology, and genetics clinics from 2012 to 2025 for patients with urothelial cancer and a pathogenic *MSH2*, *MSH6*, *MLH1*, or *PMS2* variant, or a stated history of Lynch syndrome. Keywords including *MSH2*, *MSH6*, *MLH1*, *PMS2*, Lynch, and urothelial were queried within the Electronic Medical Record Search Engine (EMERSE) system and a subsequent chart review was performed. EMERSE is a search engine developed by University of Michigan for free-text electronic health record documents that supports efficient retrieval and standardized chart review [10]. Many patients had received testing outside of University of Michigan, and self-reported Lynch syndrome was sufficient for inclusion. Age at each cancer diagnosis was recorded. Grade and stage were categorized as metastatic for any nodal or distant disease, and were considered invasive for ≥T2. As some patients had recurrences and multiple urothelial sites of disease, grade and stage were reported as the highest grade and stage during available follow-up, recognizing that follow-up was shorter for patients first seen more recently and thus recurrence may not be fully captured. We also recorded the number of preceding major noncutaneous cancer resections for these patients. This was IRB approved through University of Michigan (HUM00226722).

All other statistics, besides our comparison of bladder cancer only and UTUC, were descriptive, with reporting of medians and interquartile ranges for all continuous variables, and counts and percentages for all categorical variables. Where timing of cancer diagnosis was needed, as for swimmer plots and for reporting of a preceding cancer history, only patients with age at each cancer diagnosis were included. Analysis and figures were made in R version 4.5.1 (R Foundation for Statistical Computing, Vienna, Austria) using the packages *ggplot2*, *cowplot*, and *dplyr*.

## 3 Results

### 3.1 Prevalence of germline variants in a cohort referred for testing

Within the Myriad cohort, we identified 3561 patients with a personal history of urothelial cancer who received germline testing; this included 3130 patients with bladder cancer, 370 with UTUC, and 61 with both bladder cancer and UTUC. Clinical characteristics of this cohort are presented in Table 1.

**Table 1:** Cohort characteristics

Characteristic	Bladder cancer only (*N* = 3130) a	Upper tract urothelial cancer b (*N* = 431) a
Sex		
Female	1956 (62)	273 (63)
Age at urothelial cancer diagnosis	60 (50–69)	58 (51–66)
Urothelial cancer site		
Bladder	3130 (100)	61 (14)
Ureter	0	272 (63)
Renal pelvis	0	181 (42)
Family history of urothelial cancer	347 (11)	54 (13)
Self-reported ancestry		
Ashkenazi Jewish	170 (5.4)	21 (4.9)
Asian	43 (1.4)	6 (1.4)
Black/African	102 (3.3)	14 (3.2)
Central/Eastern Europe	125 (4.0)	20 (4.6)
Hispanic/Latino	77 (2.5)	19 (4.4)
Middle Eastern	14 (0.4)	1 (0.2)
Native American	57 (1.8)	7 (1.6)
None specified	403 (13)	62 (14)
Other	53 (1.7)	6 (1.4)
Pacific Islander	1 (<0.1)	0
Western/Northern Europe	684 (22)	148 (34)
White/Non-Hispanic	1401 (45)	127 (29)
Germline panel c		
Broad	2112 (69)	232 (55)
Targeted	961 (31)	193 (45)
Unknown	57	6

UTUC = upper tract urothelial carcinoma.

a Data are presented as *n* (%) or median (Q1–Q3).

b Of the 431 patients with UTUC, 61 also had reported bladder cancer.

c Panel was considered broad if nine or more genes were tested and targeted if the number of genes was <9. The exact genes tested were not known for 63 patients.

Overall, 702 (20% of tested patients) patients had pathogenic or likely pathogenic variants, including 618 with pathogenic variants and 84 with likely pathogenic variants. All variants were classified as per the guidelines by the American College of Medical Genetics and Genomics/Association for Molecular Pathology. Among 84 likely pathogenic variants, 83 (99%) were concordant, pathogenic, or likely pathogenic in ClinVar.

One variant was classified as of uncertain significance on corroboration in ClinVar—BRCA1 c.40G>T (p.Val14Phe); however, functional data indicated loss of function, and this patient was retained for the analysis [8], [11].

**Fig. 1:**
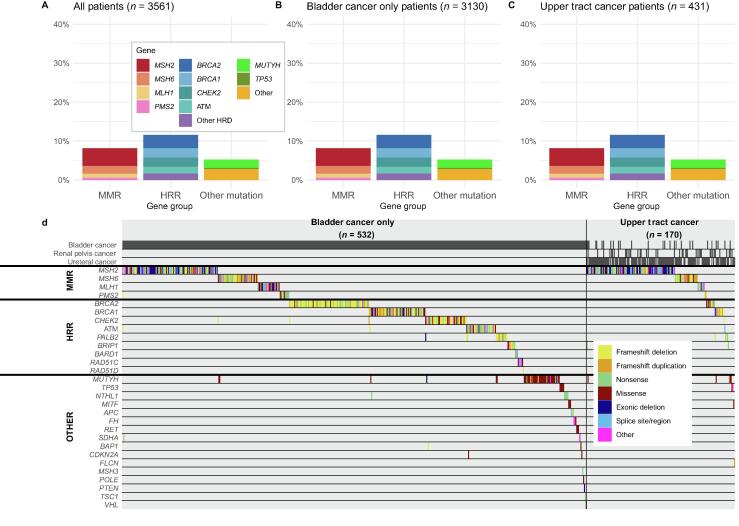
Pathogenic and likely pathogenic variants among patients with urothelial cancer referred for germline testing, reported for (A) all patients, (B) patients with bladder cancer only, and (C) patients with upper tract urothelial cancer. (D) A tile plot showing only the germline pathogenic and likely pathogenic variants identified, divided by patients with bladder cancer only and upper tract urothelial cancer. HRD = homologous recombination deficiency; HRR = homologous recombination repair; MMR = mismatch repair.

A total of 328 patients were identified with germline variants in an MMR gene, consistent with Lynch syndrome (Fig. 1 and Supplementary Table 1). This included 210 patients with *MSH2* variants, 72 with *MSH6* variants, 33 with *MLH1* variants, and 14 with *PMS2* variants. One patient had a deletion in *MSH2* and a frameshift variant in *PMS2*. In all, 298 patients were identified with variants in HRR genes, including 101 with *BRCA2*, 75 *BRCA1*, 51 *CHEK2*, 37 *ATM*, 20 *PALB2*, and 21 other HRR genes (*BRIP1*, *BARD1*, *RAD51C*, and *RAD51D*). Three patients had both MMR and HRR variants (*PMS2* + ATM, *MSH6* + *CHEK2*, and *MSH2* + *BRIP1*), and seven patients had two HRR variants (combinations of *BRCA1, BRCA2, CHEK2, ATM,* and *PALB2*).

Consistent with its prevalence in the general population, a moderate percentage of *MUTYH* carriers (2.1%, *n* = 53) were detected in the cohort [12]. Other variants, outside of MMR and HRR, were rare, including only seven *TP53* variants.

When examined separately, 27% (*n* = 101) of the UTUC patients tested positive for an *MSH2* variant, in contrast with only 4.6% (*n* = 109) of bladder cancer–only patients (*p* < 0.001). Of the UTUC patients, 7.7% (*n* = 26) and 2.2% (*n* = 8) had an *MSH6* and an *MLH1* variant, respectively, in comparison with 2.0% (*n* = 46) and 1.1% (*n* = 25) in patients with bladder cancer only (*p* < 0.001 and *p* = 0.068, respectively). Overall, 32% (*n* = 137) of all UTUC patients and 6.1% (*n* = 191) of all bladder cancer–only patients were found to have an MMR variant. Other non-MMR variants were much more similarly distributed between UTUC and bladder cancer patients. For instance, 3.7% (*n* = 10) and 3.4% (*n* = 91) of tested UTUC and bladder cancer–only patients had *BRCA2* variants, respectively.

### 3.2 Personal and family cancer histories of urothelial cancer patients with germline MMR and HRR variants

**Fig. 2:**
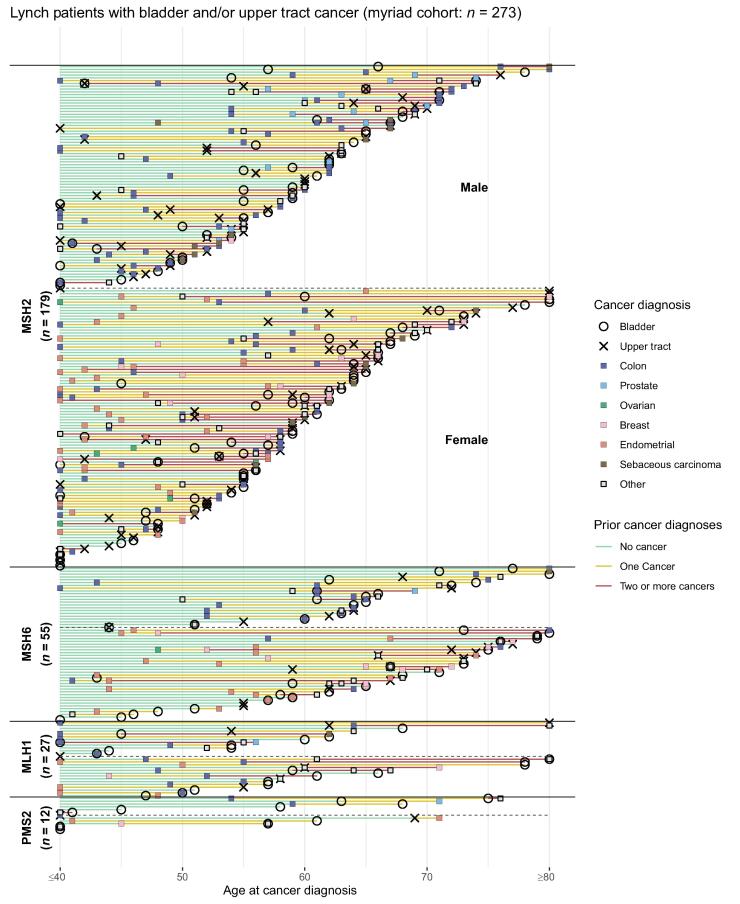

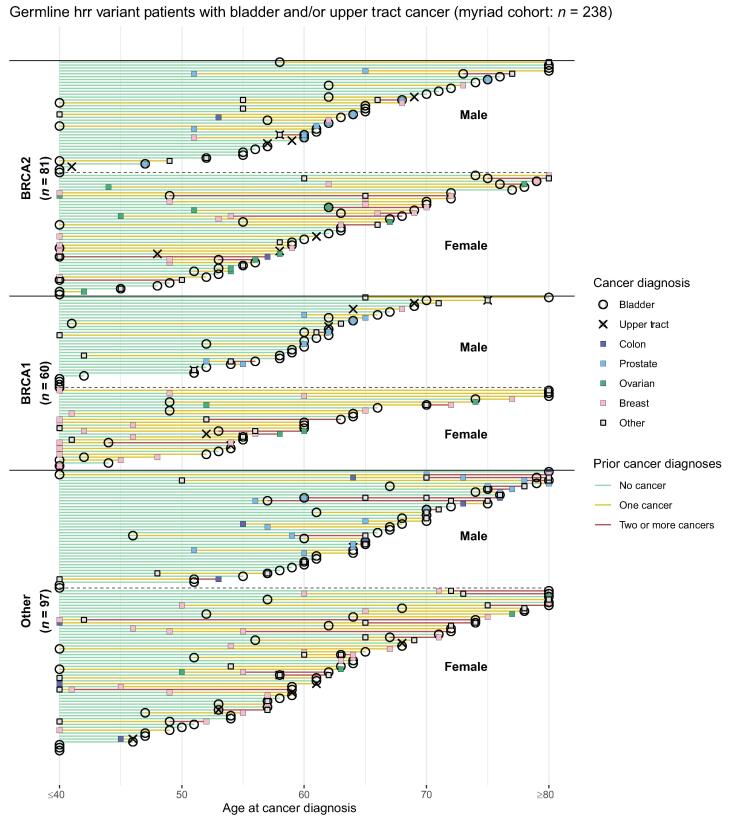

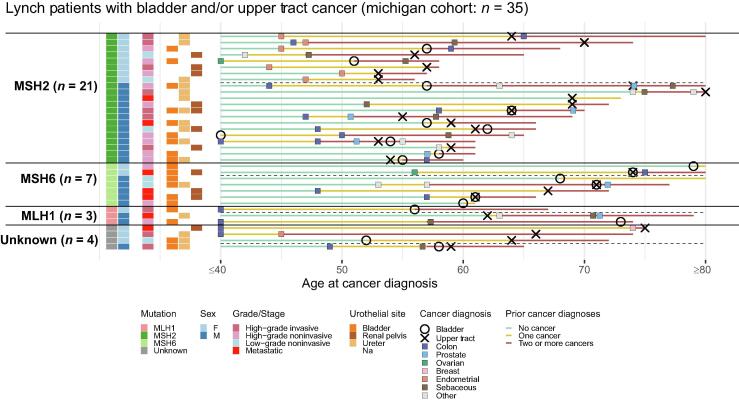
Swimmer plots for personal cancer history among the Myriad cohort of patients with (Aa) Lynch and urothelial cancer (*n* = 273) and (B) germline HRR and urothelial cancer (*n* = 296). Each event represents the age at the first diagnosis of a given cancer. Line ends at last cancer diagnosis in registry. (C) Personal cancer history in the Michigan cohort (*n* = 35) among patients with Lynch syndrome and urothelial cancer. Line ends at censoring or death. F = female; HRR = homologous recombination repair; M = male; NA = not available.

Within the Myriad cohort, many patients with Lynch syndrome had a cancer diagnosis preceding their urothelial cancer diagnosis, including 63% of patients (*n* = 112) with an *MSH2* variant, 66% (*n* = 33) with an *MSH6* variant*,* 59% (*n* = 16) with an *MLH1* variant, and 53% (*n* = 28) with an *MLH1* variant (Table 2 and Fig. 2A)*.* Colon cancer was the most common nonurothelial cancer seen, including 60% (*n* = 126) of patients with *MSH2.* Endometrial cancer was also observed commonly, including 41% (*n* = 47) of female patients with *MSH2.* The median age of urothelial carcinoma diagnosis ranged from 57 to 68 yr depending on the variants, only slightly younger than in the general population.

**Table 2:** Personal cancer histories among patients with urothelial cancer and either a germline pathogenic/likely pathogenic variant in MMR or HRR genes, or no germline variants identified a, b, c

			MMR variants				HRR variants			No germline variant identified d
			*MSH2* (*n* = 210)	*MSH6* (*n* = 72)	*MLH1* (*n* = 33)	*PMS2* (*n* = 14)	*BRCA2* (*n* = 101)	*BRCA1* (*n* = 75)	Non-*BRCA* HRR e (*n* = 126)	(*n* = 2174)
	Sex									
	Female, (*n*)		55 (116)	57 (41)	48 (16)	43(6)	50 (51)	48 (36)	53 (67)	66 (1427)
	Age at urothelial cancer diagnosis		56 (48.5–64)	65 (59–72.5)	57.5 (50.75–63)	57.5 (42–66)	61 (53–69.5)	58 (51–64)	61.5 (54–70)	60 (50–68)
	Age at bladder cancer diagnosis		**58** **(50–64.25)**	**65** **(59–72.75)**	57 (50–66)	57.5 (44–64)	62 (53–70)	59 (51–64)	*61.5 (50–68.75)*	60 (50–68.75)
	Age at upper tract urothelial cancer diagnosis		55 (48–64)	66 (60–72)	58 (54.5–61)	54.5 (47.25–61.75)	58 (57–61)	54.5 (51.25–63.5)	60 *(56.75–65)*	59 (53–66)
	Number of cancer diagnoses preceding urothelial cancer e									
	0		37 (67)	34 (22)	41 (11)	46 (6)	65 (53)	62 (37)	59 (58)	60 (1093)
	1		41 (73)	38 (25)	48 (13)	38 (5)	32 (26)	32 (19)	27 (27)	31 (553)
	≥2		22 (39)	12 (8)	11 (3)	15 (2)	2.5 (2)	6.7 (4)	14 (14)	9.2 (167)
	Urothelial cancer	Any urothelial	100 (210)	100 (72)	100 (33)	100 (14)	100 (101)	100 (75)	100 (126)	100 (2174)
		Bladder	60 (126)	69 (50)	76 (25)	86 (12)	92 (93)	89 (67)	95 (120)	92 (2006)
		Renal pelvis	10 (21)	10 (7)	12 (4)	0 (0)	4.0 (4)	2.7 (2)	1.6 (2)	4.8 (104)
		Ureter	41 (86)	28 (20)	12 (4)	14 (2)	5.9 (6)	11 (8)	4.8 (6)	4.8 (104)
	MMR associated	Colon	60 (126)	47 (34)	55 (18)	21 (3)	3.0 (3)	0 (0)	9.5 (12)	12 (265)
	-	Endometrial e	41 (47)	49 (20)	44 (7)	33 (2)	3.9 (2)	5.6 (2)	9.0 (6)	8.7 (124)
	-	Sebaceous	13 (27)	5.6 (4)	6.1 (2)	0 (0)	0 (0)	0 (0)	0 (0)	0.3 (6)
HRR associated	-	Ovarian e	8.6 (10)	4.9 (2)	6.2 (1)	0 (0)	22 (11)	17 (6)	10 (7)	9.3 (133)
-	-	Prostate e	21 (20)	6.5 (2)	18 (3)	13 1	28 (14)	18 (7)	47 (28)	37 (275)
-		Breast e	18 (22)	29 (12)	19 (4)	17 (1)	57 (33)	67 (25)	64 (43)	48 (748)

HRR = homologous recombination repair; IQR = interquartile range; MMR = mismatch repair.

a Data are presented as % (*n*) or median (IQR).

b Non-BRCA HRR variants included *CHEK2* (*n* = 51), *ATM* (*n* = 37), *PALB2* (*n* = 20), *BRIP1* (*n* = 11), *BARD1* (*n* = 3), *RAD51C* (*n* = 6), and *RAD51D* (*n* = 1). Three patients had two variants in non-*BRCA* HRR genes.

c Percentages for endometrial, breast, and, ovarian cancers reported among female patients, and percentages for prostate cancer reported among male patients. There were one, one, four, one, three, and 56 male patients with breast cancer with *MSH2*, *MLH1*, *BRCA2*, *BRCA1*, non-*BRCA* HRR, and no germline variants, respectively.

d Among tested patients without pathogenic/likely pathogenic or variants of uncertain significance in all tested genes.

e Of patients without missing data on diagnosis year.

A family history of urothelial cancer was not common in this cohort, whereas a family history of colon cancer was very common (Table 3). Despite being a highly selected cohort of patients with Lynch syndrome and urothelial cancer, only 22% (*n* = 47) of those with *MSH2* variants reported a family history of bladder cancer or UTUC, compared with 80% (*n* = 167) reporting a family history of colon cancer and 31% (*n* = 65) of endometrial cancer. The family histories of colon and endometrial cancers were often found in a first-degree relative. For instance, 70% (*n* = 147) of patients with *MSH2* had a parent, child, or sibling with colon cancer (Supplementary Table 2).

**Table 3:** Family cancer histories among patients with urothelial cancer and either a germline pathogenic/likely pathogenic variant identified in MMR or HRR genes, or no germline variants identified a

		Family history of a given cancer	Family cancer history							
			MMR variants				HRR variants			No germline variant identified b
			*MSH2* (*n* = 210)	*MSH6* (*n* = 72)	*MLH1* (*n* = 33)	*PMS2* (*n* = 14)	*BRCA2* (*n* = 101)	*BRCA1* (*n* = 75)	Non-*BRCA* HRR c (*n* = 126)	(*n* = 2174)
	Urothelial cancer	Any urothelial	22 (47)	17 (12)	9.1 (3)	29 (4)	5.0 (5)	6.7 (5)	13 (17)	11 (231)
		Bladder	15 (31)	11 (8)	9.1 (3)	21 (3)	4.0 (4)	6.7 (5)	13 (17)	10 (220)
		Renal pelvis	1.9 (4)	0 (0)	0 (0)	(0)	(0)	(0)	(0)	0.4 (8)
		Ureter	6.7 (14)	5.6 (4)	3.0 (1)	7.1 (1)	1.0 (1)	1.3 (1)	(0)	0.3 (7)
	MMR associated	Colon	8 (167)	65 (47)	79 (26)	86 (12)	20 (20)	16 (12)	29 (36)	29 (623)
	-	Endometrial	31 (65)	31 (22)	12 (4)	14 (2)	5.0 (5)	6.7 (5)	9.5 (12)	8.5 (184)
	-	Sebaceous	3.3 (7)	(0)	3.0 (1)	(0)	(0)	(0)	0.8 (1)	0.0 (1)
HRR associated	-	Ovarian	15 (32)	15 (21)	18 (6)	21 (3)	32 (32)	41 (31)	11 (14)	17 (374)
-	-	Prostate	15 (32)	14 (10)	12 (4)	43 (6)	18 (18)	9.3 (7)	33 (42)	17 (373)
-		Breast	23 (49)	38 (27)	27 (9)	57 (8)	75 (76)	80 (60)	58 (73)	55 (1198)

HRR = homologous recombination repair; MMR = mismatch repair.

a Data are presented as % (*n*).

b Excluding both pathogenic/likely pathogenic and variants of uncertain significance in all tested genes.

c Non-*BRCA* HRR variants included *CHEK2* (*n* = 51), *ATM* (*n* = 37), *PALB2* (*n* = 20), *BRIP1* (*n* = 11), *BARD1* (*n* = 3), *RAD51C* (*n* = 6), and *RAD51D* (*n* = 1). Three patients had two variants in non-*BRCA* HRR genes.

Many of the patients with a germline HRR variant also had a preceding history of cancer (Table 2 and Fig. 2B). This ranged from 35% (*n* = 28) in *BRCA2* to 41% (*n* = 41) in non-*BRCA* HRR variants (Table 2). The most common other malignancy was breast cancer, which was observed in 57% (*n* = 33) of female patients with *BRCA2* and 67% (*n* = 25) of female patients with *BRCA1* variants*.* Prostate cancer was also common, seen in 28% (*n* = 14) of male patients with *BRCA2* and 51% (*n* = 7) with *BRCA1* variants*.*

Breast cancer was the most common cancer in the family history of urothelial cancer patients with HRR germline variants and was observed in 75% (*n* = 76) of patients with *BRCA1* and 80% (*n* = 60) of patients with *BRCA2* (Table 3)*.* This was often seen in a first-degree relative—63% (*n* = 47) of patients with *BRCA1* and 59% (*n* = 60) with *BRCA2.*

Swimmer plots for Figures 2A and 2B were based on patients in whom complete ages at each cancer diagnosis were available. This included 273 of the 328 patients with MMR variants and 238 of the 298 patients with HRR variants. Any patients with missing ages for a cancer diagnosis were excluded from these figures. Of the 3561 patients included, 644 did not have complete ages at cancer diagnosis for their entire cancer histories and were excluded from the swimmer plots. They were included in all other analyses.

The percentages of patients who would meet the germline testing criteria put forth by the EAU UTUC guidelines are described in Supplementary Table 4. The criteria are a modified version of the Amsterdam II criteria [13]. Given the absence of specific guidelines for bladder cancer, these guidelines were also applied to the bladder cancer–only cohort, with the understanding that these guidelines are not currently being applied to this population. Of the entire cohort, 46% (*n* = 152) of patients would meet screening criteria by age (<60 yr old) and 46% (*n* = 152) would meet screening criteria by a preceding Lynch spectrum cancer (defined within the guidelines as colorectal, endometrial, or upper gastrointestinal tract cancer). Combined, 75% of Lynch syndrome patients with urothelial cancer (*n* = 247) would meet one of these two criteria. When a first-degree relative <50 yr old or two first-degree relatives with a Lynch spectrum cancer were also included as the criteria, this increased to 87% (*n* = 286).

The ancestry of patients with MMR and HRR variants is shown in Supplementary Table 5, and a higher prevalence of Ashkenazi Jewish ancestry was observed in *BRCA2* and *BRCA1* carriers than in the carriers of other variants. The ordering providers associated with the Myriad tests are shown in Supplementary Table 5. The largest portion of tests was ordered by medical oncology providers (*n* = 1533), with very few tests ordered by urologists (*n* = 128).

### 3.3 Michigan external comparison cohort

To corroborate our Lynch syndrome findings from the Myriad cohort, we identified 35 patients with urothelial cancer and a history of Lynch syndrome at University of Michigan. This cohort included 21 patients with *MSH2*, seven patients with *MSH6*, three patients with *MLH1*, and four patients in whom the exact variant was not known. In this cohort, 19 patients underwent nephroureterectomy, four underwent cystectomy, and three underwent segmental ureterectomy. Of these patients, 20% (*n* = 7) had metastatic urothelial cancer, 34% (*n* = 12) had high-grade invasive disease, 29% (*n* = 10) had high-grade noninvasive disease, and 17% (*n* = 6) had low-grade noninvasive disease.

Consistent with our findings in the Myriad cohort, many (74%, *n* = 26) patients in the Michigan cohort had a preceding cancer diagnosis (Fig. 2C and Supplementary Table 3). Of the patients, 54% had a history of colon cancer and 50% of female patients had a history of endometrial cancer.

Of the Michigan patients with Lynch syndrome, 68% (*n* = 24) had undergone a major cancer resection prior to their urothelial cancer diagnosis, with 30 total surgeries (Supplementary Table 3). In order of frequency, this included 13 colectomies, nine hysterectomies/salpingo-oophorectomies, two rectal cancer resections, one mastectomy, one adrenalectomy, one hepatectomy, one nephrectomy, one Whipple, and one prostatectomy.

A subset of patients in both the Myriad and the Michigan Lynch syndrome cohorts had sebaceous carcinomas, a skin cancer highly specific for Lynch syndrome and eponymously denoted as Muir-Torre syndrome; this was observed in with 13% (*n* = 27) and 38% (*n* = 8) of *MSH2* carriers in the Myriad and Michigan cohorts, respectively.

## 4 Discussion

This study confirms the high prevalence of Lynch syndrome in patients with UTUC, with over 30% of those referred for testing having pathogenic MMR variants, most often MSH2 [4], [14]. Despite this high prevalence, bladder cancer was still more frequently observed than UTUC among patients with Lynch syndrome, with ∼60% of patients with Lynch syndrome in both the Myriad and the Michigan cohort presenting with bladder cancer. These findings, consistent with those of prior series, suggest that restricting germline testing to UTUC alone would miss a substantial proportion of Lynch syndrome–associated bladder cancer cases [4], [15].

Many patients with Lynch syndrome had a cancer diagnosis prior to their urothelial cancer, most often colon cancer (≥50%) or endometrial cancer in female patients (≥40%). In the Michigan cohort, 68% had undergone a major cancer resection, most frequently colectomy or hysterectomy, before their urothelial cancer diagnosis. The median age of urothelial cancer diagnosis in patients with Lynch syndrome in our cohort was 59 yr, older than the age of diagnosis of other cancers in Lynch syndrome patients and consistent with the median age of onset for urothelial cancer in the Prospective Lynch Syndrome cohort, which was between 61 and 67 yr depending on the affected gene, compared with between 49 and 54 yr for colorectal, endometrial, and ovarian cancers [4]. Although penetrance for urothelial cancer is much lower than for colon or endometrial cancer in patients with Lynch syndrome, the gap narrows as patients age. For instance, it is approximately ten-fold lower at age 50 yr and only two- to three-fold lower by age 75 yr among MSH2 carriers. As many Lynch syndrome–associated urothelial cancer cases occur after the age of 60 yr, age-based criteria alone risk missing the majority of carriers. This underscores the importance of personal and family histories in guiding testing.

The role of germline HRR variants in urothelial cancer is less clear, with putative associations with *BRCA2*, *BRCA1*, *ATM*, and *BRIP1* [16], [17], [18], [19]. In this cohort, patients with HRR variants had a median urothelial cancer diagnosis age of 60 yr, and 30–40% had a preceding cancer diagnosis, most often prostate or breast cancer. A family history of breast cancer was similarly common. These findings reinforce that personal and family cancer histories should inform germline testing decisions for both Lynch and HRR genes. Expansion of therapeutic options with PARP inhibition for HRR cancers, particularly for those with *BRCA1* and *BRCA2* variants, further underscores the importance of identifying HRR carriers [6], [20].

As Lynch syndrome outcomes for colorectal, endometrial, and ovarian cancers improve with surveillance and early intervention, the importance of urothelial cancer in Lynch syndrome patients has grown. For instance, in the Prospective Lynch Syndrome cohort, in MSH2 carriers who underwent regular colonoscopy, 20 deaths were attributed to colon cancer and 19 to urothelial cancer (11 UTUC and eight bladder cancer) [10]. This shift highlights a growing unmet need: as traditional Lynch syndrome–associated cancers are managed better, urothelial malignancies will account for a larger share of morbidity and mortality, and better protocols for urinary tract monitoring will be needed.

Guidelines currently recommend universal MMR immunohistochemistry or MSI screening of UTUC tumor specimens—since 2022 by the EAU and 2023 by the American Urological Association (AUA)—with germline testing pursued in the setting of MMR loss or MSI-high status [9], [21]. This approach mimics longstanding recommendations in endometrial and colorectal cancers for MMR staining; yet, real-world uptake in these cancers remains inconsistent [22], [23], and not all patients with MMR-deficient tumors are referred appropriately for germline evaluation [23]. As germline testing becomes less costly, more covered by insurance, and more familiar to urologists and oncologists, broader germline testing in UTUC, not just as a reflex test for MMR loss, is appropriate. While tumor-based MMR immunohistochemistry screening should remain standard, it should not serve as a prerequisite or barrier to germline testing in patients with UTUC, especially in those with a suggestive personal or family history.

Currently, both the EAU and the AUA guidelines for UTUC rely on the Amsterdam II criteria for consideration of germline testing outside of tumor-based screening. In our cohort, 9% of UTUC patients with confirmed Lynch syndrome would not have met the EAU criteria for germline testing. When relying solely on age and personal cancer history, 24% of Lynch syndrome carriers would have been missed (Supplementary Table 4). Among patients with bladder cancer only, application of these revised Amsterdam II criteria would have referred 86% of Lynch syndrome carriers for testing. Importantly, no current guidelines specify which patients with bladder cancer should undergo germline testing; application of the EAU UTUC Revised Amsterdam II framework to bladder cancer represents a reasonable interim approach, provided that clinicians give particular attention to family history when identifying at-risk individuals (Supplementary Table 2) [9].

Universal germline testing in patients with urothelial cancer may serve as one practical strategy to overcome the limitations of reflex tumor-based approaches and reliance on provider-driven selection. Our findings demonstrate that many patients with Lynch syndrome present with bladder-only disease and, though most have suggestive cancer histories, many have a prior cancer history that may not trigger testing under current guidelines. Furthermore, referral patterns in our cohort varied by specialty and were likely often influenced by other prior malignancies rather than the urothelial cancer diagnosis itself. As costs decrease and the clinical utility of identifying germline variants grows, including for cascade testing, surveillance, and especially therapeutic implications for those with Lynch syndrome, universal testing offers a streamlined, equitable approach that can improve identification of hereditary cancer syndromes and reduce disparities in access to genetic evaluation. Identification of a pathogenic variant also has immediate relevance for first-degree relatives, enabling cascade testing and cancer-specific surveillance.

Finally, there is an established and growing role of immune checkpoint inhibitors (ICIs) in tumors with MMR deficiency, including both germline and somatic alterations. This includes a tissue-agnostic indication for pembrolizumab in metastatic MMR-deficient solid tumors and a growing role of ICIs in organ preservation for localized MMR-deficient solid tumors, including in urothelial cancer [2], [24], [25]. Lynch syndrome is a highly actionable diagnosis with direct therapeutic implications, and missed identification represents a lost opportunity to guide ICI eligibility and improve individual patient outcomes directly.

We observed a predominance of ureteral over renal pelvis cancers in UTUC patients with Lynch syndrome, in contrast to sporadic UTUC where renal pelvis cancers predominate. This pattern, noted by other groups, may reflect distinct biological mechanisms of urothelial carcinogenesis in Lynch syndrome [14]. In colon cancer, immunosurveillance is believed to control and eliminate many early MMR-deficient clones [26], [27]. One hypothesis is that the ureter may represent an immune-excluded reservoir of urothelium where early clones with MMR loss can evade immunosurveillance. To our knowledge, there has not been any translational work on the predominance of ureteral cancer in Lynch syndrome.

The generalizability of our study is limited by a highly selected cohort of patients referred for germline testing, which is not reflective of the broader urothelial cancer population. Many patients were younger and had multiple cancers, and the urothelial cancer diagnosis may not have been the indication for germline testing. As such, the variant prevalence in our cohort is higher than in an unselected population. Smoking history, grade, stage, and histology were unavailable in the registry dataset, precluding assessment of tobacco interactions or disease phenotype; we attempted to mitigate this through the Michigan external comparison cohort, where a chart review provided more detailed annotation. Other referral-related factors that affect this cohort include variable panel size, a nearly 30-yr testing span, and an enrichment for women, reflecting stronger testing recommendations for breast and gynecological cancers. Indeed, after medical oncologists, gynecologists were the second most common ordering providers in this cohort (Supplementary Table 5). Only 39 Lynch syndrome carriers in this cohort had isolated urothelial cancer, without additional malignancies before or after diagnosis, representing 6.6% of the 593 patients who had germline testing for MMR with an isolated urothelial cancer. Given the absence of explicit germline testing guidelines for urothelial cancer, this number is highly subject to a selection bias and less reflective of true population penetrance.

Despite these limitations, this remains the largest descriptive cohort of urothelial cancer patients referred for germline testing, including a substantial UTUC subset. Our results are largely consistent with those of prior retrospective and prospective series in urothelial cancer [16], [28], [29]. For instance, within Memorial Sloan Kettering, 586 patients with urothelial cancer, including 86 patients with UTUC, were prospectively tested. Twelve germline Lynch syndrome variants (eight *MSH2*, two *MSH6*, and two *MLH1*) were identified, ten of which were in patients with UTUC, and nine and eight *BRCA2 and BRCA1* variants, respectively, were identified [28]. Our analysis expands on this work by offering a more detailed description of the personal and family cancer histories of patients with germline MMR and HRR variants, thus offering unique insights into the landscape of germline variants in urothelial cancer.

## 5 Conclusions

Lynch syndrome is highly prevalent in patients with UTUC; yet, many carriers present with urothelial cancer of the bladder only, who would be missed under current guideline-based testing. Personal and family histories of Lynch syndrome– or HRR-associated cancers are common in carriers and often precede the diagnosis of urothelial cancer, underscoring the value of a thorough clinical history in guiding genetic evaluation. These findings support routine germline testing for patients with UTUC and raise consideration for universal testing in urothelial cancer patients more broadly. At the very least, germline testing should be pursued in any patient with a suggestive personal or family history, regardless of whether the urothelial tumor arises in the bladder or upper tract.

***Author contributions*:** Steven M. Monda had full access to all the data in the study and takes responsibility for the integrity of the data and the accuracy of the data analysis.

*Study concept and design*: Monda, Oh, Lewicki, Else, Nguyen, Schiewer, Finch, Salami, Stoffel, Morgan, Chandrasekar, Singhal.

*Acquisition of data*: Monda, Schiewer, Finch, Singhal, Chandrasekar.

*Analysis and interpretation of data*: Monda, Oh, Morgan, Singhal, Morgan, Chandrasekar, Singhal, Else, Reichert.

*Drafting of the manuscript*: Monda, Oh, Lewicki, Kaffenberger, Else, Ghani, Nguyen, Reichert, Tsung, Humble, Schiewer, Finch, Salami, Stoffel, Morgan, Chandrasekar, Singhal.

*Critical revision of the manuscript for important intellectual content*: Monda, Oh, Lewicki, Kaffenberger, Else, Ghani, Nguyen, Reichert, Tsung, Humble, Schiewer, Finch, Salami, Stoffel, Morgan, Chandrasekar, Singhal.

*Statistical analysis*: Monda.

*Obtaining funding*: Monda, Morgan.

*Administrative, technical, or material support*: Monda, Morgan, Singhal.

*Supervision*: Singhal, Morgan.

*Other*: None.

***Financial disclosures:*** Steven M. Monda certifies that all conflicts of interest, including specific financial interests and relationships and affiliations relevant to the subject matter or materials discussed in the manuscript (eg, employment/affiliation, grants or funding, consultancies, honoraria, stock ownership or options, expert testimony, royalties, or patents filed, received, or pending), are the following: Rob Finch and Matthew Schiewer are employees of Myriad Genetics. Todd Morgan has a consulting or advisory role with ClevelandDx, Foundation Medicine, Merck, Stratify Genomics, and Tempus; research funding—MDxHealth (Inst). Samuel Kaffenberger has a consulting or advisory role with Clovis Oncology, MDxHealth, and Pfizer and has received travel, accommodations, and expenses from BMS. Simpa Salami has a consulting or advisory role with Bayer; and patents, royalties, and other intellectual property including a patent filed for 15G Score (Inst). The remaining authors have nothing to disclose.

***Funding/Support and role of the sponsor*:** None.
