# Supplementary material for: Germline Variants in Bladder and Upper Tract Urothelial Cancers: Prevalence and Clinical Context in a Large Testing Registry
Source: Eur Urol Open Sci. 2026 Feb 23;86:56–67. doi: 10.1016/j.euros.2026.02.010 (PMC13080474; doi:10.1016/j.euros.2026.02.010)
Supplement: Supplementary Data 1 [file mmc1.docx]

**Supplementary Table 1:** Pathogenic and likely pathogenic variants identified among all patients, patient with urothelial cancer of the bladder only, and patients with UTUC. Reported as the number of positive tests divided by the number of patients in whom each gene was tested.

|  |  | All Patients  (n=3561) | | | Bladder Cancer Only Patients  (n=3130) | | | UTUC Patients (n=431) | | |  |
| --- | --- | --- | --- | --- | --- | --- | --- | --- | --- | --- | --- |
|  |  | % | positive tests | # of tests | % | positive tests | # of tests | % | positive tests | # of tests | p-value* |
| MMR | *MSH2* | 7.6% | 210 | 2759 | 4.6% | 109 | 2382 | 26.8% | 101 | 377 | <0.00001 |
|  | *MSH6* | 2.7% | 72 | 2678 | 2.0% | 46 | 2340 | 7.7% | 26 | 338 | <0.00001 |
|  | *MLH1* | 1.2% | 33 | 2723 | 1.1% | 25 | 2365 | 2.2% | 8 | 358 | 0.068 |
|  | *PMS2* | 0.6% | 14 | 2507 | 0.5% | 12 | 2224 | 0.7% | 2 | 283 | 0.67 |
| HRR | *BRCA2* | 3.4% | 101 | 2954 | 3.4% | 91 | 2682 | 3.7% | 10 | 272 | 0.73 |
|  | *BRCA1* | 2.5% | 75 | 2944 | 2.4% | 65 | 2674 | 3.7% | 10 | 270 | 0.22 |
|  | *CHEK2* | 2.2% | 51 | 2349 | 2.4% | 50 | 2117 | 0.4% | 1 | 232 | - |
|  | *ATM* | 1.6% | 37 | 2349 | 1.7% | 36 | 2117 | 0.4% | 1 | 232 | - |
|  | *PALB2* | 0.9% | 20 | 2350 | 0.8% | 16 | 2118 | 1.7% | 4 | 232 | - |
|  | *BRIP1* | 0.5% | 11 | 2344 | 0.4% | 9 | 2112 | 0.9% | 2 | 232 | - |
|  | *BARD1* | 0.1% | 3 | 2343 | 0.1% | 3 | 2111 |  | 0 | 232 | - |
|  | *RAD51C* | 0.3% | 6 | 2344 | 0.3% | 6 | 2112 |  | 0 | 232 | - |
|  | *RAD51D* | 0.0% | 1 | 2344 | 0.0% | 1 | 2112 |  | 0 | 232 | - |
| Other | *MUTYH* | 2.1% | 53 | 2468 | 2.2% | 48 | 2211 | 1.9% | 5 | 257 | - |
|  | *TP53* | 0.3% | 7 | 2347 | 0.2% | 5 | 2115 | 0.9% | 2 | 232 | - |
|  | *NTHL1* | 0.4% | 6 | 1572 | 0.4% | 6 | 1423 |  | 0 | 149 | - |
|  | *MITF* | 0.4% | 4 | 914 | 0.4% | 3 | 810 | 1.0% | 1 | 104 | - |
|  | *APC* | 0.1% | 3 | 2383 | 0.1% | 3 | 2147 |  | 0 | 236 | - |
|  | *FH* | 0.3% | 3 | 914 | 0.4% | 3 | 810 |  | 0 | 104 | - |
|  | *RET* | 0.3% | 3 | 914 | 0.4% | 3 | 810 |  | 0 | 104 | - |
|  | *SDHA* | 0.3% | 3 | 914 | 0.4% | 3 | 810 |  | 0 | 104 | - |
|  | *BAP1* | 0.2% | 2 | 914 | 0.2% | 2 | 810 |  | 0 | 104 | - |
|  | *CDKN2A* | 0.1% | 2 | 2345 | 0.1% | 2 | 2112 |  | 0 | 233 | - |
|  | *FLCN* | 0.1% | 1 | 914 |  | 0 | 810 | 0.1% | 1 | 104 | - |
|  | *MSH3* | 0.1% | 1 | 1572 | 0.1% | 1 | 1423 |  | 0 | 149 | - |
|  | *POLE* | 0.0% | 1 | 2020 | 0.1% | 1 | 1831 |  | 0 | 189 | - |
|  | *PTEN* | 0.0% | 1 | 2345 | 0.0% | 1 | 2113 |  | 0 | 232 | - |
|  | *TSC1* | 0.1% | 1 | 914 | 0.1% | 1 | 810 |  | 0 | 104 | - |
|  | *VHL* | 0.1% | 1 | 914 | 0.1% | 1 | 810 |  | 0 | 104 | - |
|  | *p-value reported from Fisher’s test comparing Bladder Cancer Only Patients and UTUC Patients for *MSH2, MSH6, MLH1, PMS2, BRCA2, and BRCA1* | | | | | | | | | | |

**Supplementary Table 2:** Proportion of patients with a **first-degree family** (parent, child, or sibling) history of a given cancer among patients with urothelial cancer and either a germline pathogenic/likely pathogenic MMR/HRR variant or no germline variants identified.

|  |  |  | **MMR Variants** | | | | **HRR Variants** | | | **No Germline Variant Identified*** |
| --- | --- | --- | --- | --- | --- | --- | --- | --- | --- | --- |
|  |  | **Family History of a Given Cancer** | ***MSH2***  **(n=210)** | ***MSH6***  **(n=72)** | ***MLH1***  **(n=33)** | ***PMS2***  **(n=14)** | ***BRCA2***  **(n=101)** | ***BRCA1***  **(n=75)** | **Non-*BRCA* HRR****  **(n=126)** | **(n=2174)** |
|  | **Urothelial Cancer** | **Any Urothelial** | 18%  (37) | 14%  (10) | 6.1%  (2) | 29%  (4) | 2.0%  (2) | 6.7%  (5) | 8.7%  (11) | 7.1%  (155) |
|  |  | **Bladder Cancer** | 12.9%  (7) | 8.3%  (6) | 6.1%  (2) | 21%  (3) | 1.0%  (1) | 6.7%  (5) | 8.7%  (11) | 6.9%  (150) |
|  |  | **Renal Pelvis** | 1.4%  (3) | 0%  (0) | 0%  (0) | 0%  (0) | 0%  (0) | 0%  (0) | 0%  (0) | 0.1%  (3) |
|  |  | **Ureter** | 4.3%  (9) | 5.6%  (4) | 3.0%  (1) | 7.1%  (1) | 1.0%  (1) | 1.3%  (1) | 0%  (0) | 0.2%  (4) |
|  | **MMR Associated** | **Colon** | 70%  (147) | 47%  (34) | 73%  (24) | 43%  (6) | 6.9%  (7) | 9.3%  (7) | 18%  (23) | 18%  (393) |
|  |  | **Endometrial** | 24%  (50) | 22%  (16) | 12%  (4) | 7.1%  (1) | 5.0%  (5) | 4.0%  (3) | 7.1%  (9) | 5.1%  (110) |
|  |  | **Sebaceous Carcinoma** | 2.4%  (5) | 0%  (0) | 3.0%  (1) | 0%  (0) | 0%  (0) | 0%  (0) | 0%  (0) | 0.0%  (1) |
| **HRR Associated** |  | **Ovarian** | 10%  (21) | 11%  (8) | 15%  (5) | 14%  (2) | 22%  (22) | 35%  (26) | 4.0%  (5) | 8.0%  (175) |
|  |  | **Prostate** | 12%  (25) | 11%  (8) | 6.1%  (2) | 7%  (1) | 12%  (12) | 5.3%  (4) | 18%  (23) | 13%  (272) |
|  |  | **Breast** | 14%  (29) | 24%  (17) | 9.1%  (3) | 43%  (6) | 59%  (60) | 63%  (47) | 43%  (54) | 34%  (741) |
|  |  | *Excluding both pathogenic/likely pathogenic and variants of uncertain significance in all tested genes  **Non-BRCA HRR variants included *CHEK2* (n=51), *ATM* (n=37), *PALB2* (n=20), *BRIP1* (n=11), *BARD1* (n=3), *RAD51C* (n=6), and *RAD51D* (n=1). 3 patients had two variants in non-*BRCA* HRR genes. | | | | | | | |  |

**Supplementary Table 3:** Personal cancer history and clinical history among the Michigan cohort (n=35) of patients with Lynch and urothelial cancer.

|  |  | **MMR Variants** | | | |
| --- | --- | --- | --- | --- | --- |
|  |  | **All Patients***  **(n=35)** | ***MSH2***  **(n=21)** | ***MSH6***  **(n=7)** | ***MLH1***  **(n=3)** |
| **Sex** | |  |  |  |  |
| **Female** | | 40%  (14) | 38%  (8) | 29%  (2) | 33%  (1) |
| **Age at First Urothelial Cancer Diagnosis**  Median (IQR) | | 60  (56-68.5) | 57  (55-64) | 68  (64-72.5) | 62  (59-67.5) |
| **Age at Bladder Cancer Diagnosis** Median (IQR) | | 58  (55.75-65) | 57  (54.25-57.75) | 69.5  (62.75-73.25) | 64.5  (60.25-68.75) |
| **Age at Upper Tract Urothelial Cancer Diagnosis** Median (IQR) | | 64  (59-69) | 60  (55.75-69) | 69  (65.5-71.75) | 62  (62-62) |
| **Number of Cancer Diagnoses Preceding Urothelial Cancer**** | |  |  |  |  |
| **0** | | 26%  (9) | 19%  (4) | 43%  (3) | 33%  (1) |
| **1** | | 46%  (16) | 57%  (12) | 43%  (3) | 33%  (1) |
| **≥2** | | 29%  (10) | 24%  (5) | 14%  (1) | 33%  (1) |
| **Greatest Urothelial Cancer Grade/Stage** | |  |  |  |  |
| **Metastatic** | | 20%  (7) | 9.5%  (2) | 43%  (3) | 33%  (1) |
| **High-Grade**  **Invasive** | | 34%  (12) | 33%  (7) | 29%  (2) | 33%  (1) |
| **High-Grade**  **Non-Invasive** | | 29%  (10) | 43%  (9) | 0%  (0) | 33%  (1) |
| **Low-Grade**  **Non-Invasive** | | 17%  (6) | 14%  (3) | 29%  (2) | 0%  (0) |
| **Prior Cancer Resection** | | 69%  (24) | 71%  (15) | 57%  (4) | 66%  (2) |
| **Urothelial Cancer** | **Any Urothelial** | 100%  (35) | 100% (35) | 100%  (7) | 100%  (3) |
|  | **Bladder Cancer** | 57%  (20) | 48%  (10) | 86%  (6) | 66%  (2) |
|  | **Renal Pelvis** | 34%  (12) | 38%  (8) | 43%  (3) | 0%  (0) |
|  | **Ureter** | 46%  (16) | 52%  (11) | 14%  (1) | 33%  (1) |
|  | **Colon** | 54%  (19) | 52%  (11) | 43%  (3) | 66%  (2) |
|  | **Endometrial**** | 50%  (7) | 75%  (6) | 0%  (0) | 0%  (0) |
|  | **Sebaceous Carcinoma/Adenoma** | 31%  (11) | 38%  (8) | 0%  (0) | 66%  (2) |
|  | **Ovarian**** | 14%  (2) | 13%  (1) | 50%  (1) | 0%  (0) |
|  | **Prostate**** | 33%  (7) | 38%  (5) | 20%  (1) | 50%  (1) |
|  | **Breast**** | 7.1%  (1) | 0%  (0) | 0%  (0) | 0%  (0) |
| *Including 4 patients with self-endorsed history of Lynch on germline testing but unknown specific variants | | | | | |

**Supplementary Table 4: Proportion of Lynch Patients who would meet criteria for screening by EAU Upper Tract Urothelial Cancer (UTUC) guidelines. No existing guidelines exist for bladder cancer only patients, so the UTUC guidelines were applied to this population as well.**

| **Proposed UTUC Germline Testing Criteria*** | **UTUC Patients with Lynch**  **(n=137)** | **Bladder Cancer Only Patients with Lynch***  **(n=191)** | **All Patients with Lynch**  **(n=328)** |
| --- | --- | --- | --- |
| **Age <60 at Diagnosis** | 50%  (69) | 43% (83) | 46% (152) |
| **Personal history of a Preceding Lynch-spectrum cancer***** | 45%  (62) | 47%  (90) | 46%  (152) |
| **Any of the Above 2 Criteria** | 76%  (104) | 75%  (143) | 75%  (247) |
| **One first-degree relative <50 yr with a Lynch-spectrum cancer** | 48%  (66) | 38%  (72) | 42%  (138) |
| **Two first-degree relatives with Lynch-spectrum cancer** | 42%  (57) | 33%  (63) | 37%  (120) |
| **Any of Above 4 Criteria** | 91%  (124) | 86%  (164) | 88%  (288) |

***Based on EAU guidelines for UTUC using Modified Amsterdam II Criteria. No guidelines specifically delineate testing for bladder cancer.**

****Lynch spectrum cancer was considered as colorectal, endometrium, or small bowel. For family history, UTUC, also included.**

**Supplementary Table 5:** Self-reported ancestry of patients with germline MMR and HRR variants and no germline variants identified.

|  |  |  | **MMR Variants** | | | | **HRR Variants** | | | **No Germline Variant Identified*** | **VUS**  **Identified***** |
| --- | --- | --- | --- | --- | --- | --- | --- | --- | --- | --- | --- |
|  |  | **Ancestry** | ***MSH2***  **(n=210)** | ***MSH6***  **(n=72)** | ***MLH1***  **(n=33)** | ***PMS2***  **(n=14)** | ***BRCA2***  **(n=101)** | ***BRCA1***  **(n=75)** | **Non-*BRCA* HRR****  **(n=126)** | **(n=2174)** | **(n=685)** |
|  | **Ashkenazi Jewish** | | 4.8%  (10) | 4.2%  (3) | 0%  (0) | 0%  (0) | 21%  (21) | 13%  (10) | 3.2%  (4) | 7.0%  (153) | 5.0%  (34) |
|  | **Asian** | | 2.9%  (6) | 1.4%  (1) | 0%  (0) | 0%  (0) | 1.0%  (1) | 0%  (0) | 0.8%  (1) | 1.1%  (25) | 2.3%  (16) |
|  | **Black/African** | | 3.3%  (7) | 2.8%  (2) | 0%  (0) | 0%  (0) | 2.0%  (2) | 4.0%  (3) | 1.6%  (2) | 2.4%  (52) | 8.6%  (59) |
|  | **Central/Eastern Europe** | | 9.0%  (19) | 6.9%  (5) | 9.1%  (3) | 14%  (2) | 5.0%  (5) | 13%  (10) | 4.8%  (6) | 5.8%  (127) | 2.9%  (20) |
|  | **Hispanic/Latino** | | 3.8%  (8) | 0%  (0) | 6.1%  (2) | 7.1%  (1) | 4.0%  (4) | 0%  (0) | 0.8%  (1) | 3.2%  (69) | 3.2%  (22) |
|  | **Middle Eastern** | | 0%  (0) | 1.4%  (1) | 0%  (0) | 0%  (0) | 0%  (0) | 1.3%  (1) | 0.8%  (1) | 0.6%  (13) | 0.6%  (4) |
|  | **Native American** | | 3.8%  (8) | 1.4%  (1) | 3.0%  (1) | 0%  (0) | 2.0%  (2) | 0%  (0) | 2.4%  (3) | 3.3%  (7) | 2.2%  (15) |
|  | **None Specified** | | 14%  (30) | 17%  (12) | 21%  (7) | 7.1%  (1) | 11.9%  (12) | 13%  (10) | 11.9%  (15) | 13.5%  (294) | 11%  (77) |
|  | **Other** | | 3.8%  (8) | 1.4%  (1) | 9.1%  (3) | 7.1%  (2) | 0%  (0) | 5.3%  (4) | 2.4%  (3) | 3.2%  (70) | 2.8%  (19) |
|  | **Pacific Islander** | | 0%  (0) | 0%  (0) | 0%  (0) | 0%  (0) | 0%  (0) | 0%  (0) | 0%  (0) | 0%  (0) | 0.3%  (2) |
|  | **Western/Northern Europe** | | 52%  (109) | 38%  (27) | 55%  (18) | 21%  (3) | 26%  (26) | 40%  (39) | 16%  (20) | 29%  (632) | 10%  (71) |
|  | **White/Non-Hispanic** | | 18%  (37) | 33%  (24) | 15%  (5) | 57%  (8) | 36%  (36) | 23%  (17) | 64%  (81) | 43%  (929) | 61%  (421) |
|  |  | *Among tested patients without pathogenic/likely pathogenic or variants of uncertain significance in all tested genes  **Non-*BRCA* HRR variants included *CHEK2* (n=51), *ATM* (n=37), *PALB2* (n=20), *BRIP1* (n=11), *BARD1* (n=3), *RAD51C* (n=6), and *RAD51D* (n=1). 3 patients had two variants in non-*BRCA* HRR genes.  ***Variant of uncertain significance observed but without any observed pathogenic or likely pathogenic variants observed. | | | | | | | |  |  |

**Supplementary Table 6:** Ordering provider specialty within the Myriad cohort.

| **Specialty** | **Tests Ordered** |
| --- | --- |
| Medical Oncology | **1533** |
| Gynecology* | **524** |
| Surgery* | **503** |
| Genetics | **325** |
| Primary Care/Internal Medicine | **203** |
| Urology* | **128** |
| Gastroenterology | **111** |
| Radiation oncology | **59** |
| Other | **117** |

*****Gynecology including OB/GYN, Gynecologic Oncology, Gynecology, MFM. Surgery including surgery, colorectal surgery, and surgical oncology. Urology including urology and urologic oncology.

**Supplementary Table 7: Pathogenic or likely pathogenic variant detection rate by panel size. Proportion of patients receiving a panel that had a variant.**

|  | **Panel Size (number of genes)** | | | |
| --- | --- | --- | --- | --- |
| **Variants** | **1 - 2** | **3 - 8** | **9 - 29** | **30-49** |
| Any* | **23.4%**  **(197/839)** | **34.0% (107/315)** | **17.8%**  **(124/698)** | **15.8%**  **(260/1646)** |
| Lynch** | **35.5%**  **(53/149)** | **33.3%**  **(104/312)** | **6.9%**  **(48/698)** | **8.1%**  **(133/1646)** |
| HRR** | **15.0%**  **(98/654)** | **0%**  **(0/17)** | **9.0%**  **(63/698)** | **70**  **(4.3%)** |

* including genes outside of Lynch and HRR

**denominator only includes panels including Lynch or HRR gene respectively
